# Poly[hexa­aqua­(μ_9_-cyclo­hexane-1,2,3,4,5,6-hexa­carboxyl­ato)trimanganese(II)]

**DOI:** 10.1107/S1600536813015626

**Published:** 2013-06-15

**Authors:** Weixuan Sun, Hu Zang, Chengshi Quan

**Affiliations:** a The Class 10, 2011, Norman Bethune College of Medicine, Jilin University, 828 Xinmin Street, Changchun 130021, Jilin Province, People’s Republic of China; b Department of Orthopedics, The China–Japan Union Hospital of Jilin University, Changchun 130021, Jilin Province, People’s Republic of China; c Laboratory Teaching of Pathology, Norman Bethune College of Medicine, Jilin University, 828 Xinmin Street, Changchun 130021, Jilin Province, People’s Republic of China

## Abstract

The asymmetric unit of the title compound, [Mn_3_(C_12_H_6_O_12_)(H_2_O)_6_]_*n*_, comprises one Mn^II^ ion, one third of a cyclo­hexane-1,2,3,4,5,6-hexa­carboxyl­ate anion and two aqua ligands. The anion is completed by application of a -3 axis. The Mn^II^ ion is six-coordinated by six O atoms from two aqua ligands and three different cyclo­hexa­carboxyl­ate anions in an octa­hedral geometry. The six carboxyl­ate groups adopt a bridging bidentate mode to ligate the Mn^II^ ions. Thus, each cyclo­hexane-1,2,3,4,5,6-hexa­carboxyl­ate anion adopts a μ_9_-connected mode, ligating nine different Mn^II^ ions and forming a three-dimensional framework. In the framework, there are strong O—H⋯O hydrogen-bonding inter­actions, which further stabilize the crystal structure.

## Related literature

For background to compounds with metal-organic framework structures, see: Wang *et al.* (2010 ▶); Bourne *et al.* (2001 ▶). For their properties, uses and topologies, see: O’Keeffe *et al.* (2000 ▶); Song *et al.* (2012 ▶).
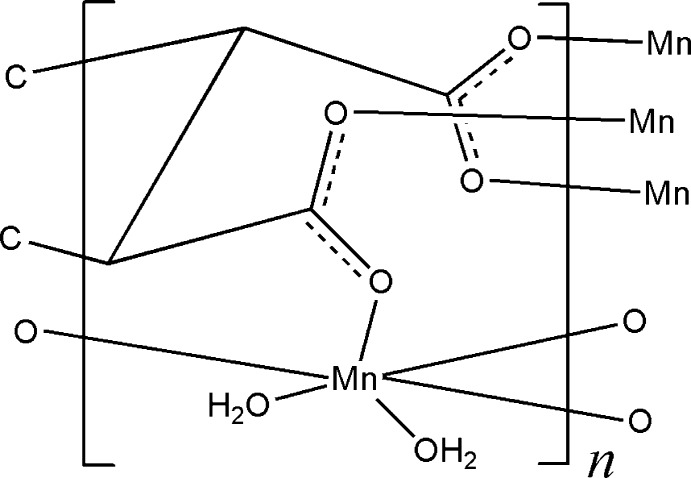


## Experimental

### 

#### Crystal data

[Mn_3_(C_12_H_6_O_12_)(H_2_O)_6_]
*M* *_r_* = 615.09
Trigonal, 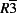

*a* = 14.5432 (4) Å
*c* = 14.9445 (10) Å
*V* = 2737.4 (2) Å^3^
*Z* = 6
Mo *K*α radiation
μ = 2.15 mm^−1^
*T* = 185 K
0.25 × 0.18 × 0.16 mm

#### Data collection

Bruker APEXII CCD diffractometer
Absorption correction: multi-scan (*SADABS*; Bruker, 2001 ▶) *T*_min_ = 0.616, *T*_max_ = 0.725
5063 measured reflections
1200 independent reflections
1098 reflections with *I* > 2σ(*I*)
*R*_int_ = 0.025

#### Refinement

*R*[*F* ^2^ > 2σ(*F* ^2^)] = 0.025
*wR*(*F* ^2^) = 0.064
*S* = 1.08
1200 reflections
112 parameters
4 restraints
H atoms treated by a mixture of independent and constrained refinement
Δρ_max_ = 0.75 e Å^−3^
Δρ_min_ = −0.28 e Å^−3^

### 

Data collection: *APEX2* (Bruker, 2007 ▶); cell refinement: *SAINT* (Bruker, 2007 ▶); data reduction: *SAINT*; program(s) used to solve structure: *SHELXTL* (Sheldrick, 2008 ▶); program(s) used to refine structure: *SHELXTL*; molecular graphics: *XP* in *SHELXTL* and *DIAMOND* (Brandenburg, 1999 ▶); software used to prepare material for publication: *SHELXTL*.

## Supplementary Material

Crystal structure: contains datablock(s) global, I. DOI: 10.1107/S1600536813015626/bx2441sup1.cif

Structure factors: contains datablock(s) I. DOI: 10.1107/S1600536813015626/bx2441Isup2.hkl

Additional supplementary materials:  crystallographic information; 3D view; checkCIF report

## Figures and Tables

**Table 1:** Hydrogen-bond geometry (Å, °)

*D*—H⋯*A*	*D*—H	H⋯*A*	*D*⋯*A*	*D*—H⋯*A*
O2*W*—H2*A*⋯O2*W* ^i^	0.81 (2)	2.31 (2)	3.116 (2)	178 (3)
O2*W*—H2*A*⋯O3^ii^	0.81 (2)	2.56 (3)	2.955 (2)	111 (2)
O1*W*—H1*B*⋯O4^iii^	0.87 (2)	1.92 (2)	2.774 (3)	169 (3)
O1*W*—H1*B*⋯O3^iii^	0.87 (2)	2.52 (3)	2.942 (3)	111 (2)
O2*W*—H2*B*⋯O1^ii^	0.84 (2)	2.06 (2)	2.883 (3)	169 (3)
O1*W*—H1*A*⋯O1*W* ^iv^	0.84 (2)	2.01 (2)	2.8513 (18)	175 (3)

Symmetry codes: (i) 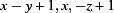; (ii) 
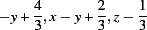
; (iii) 
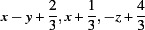
; (iv) 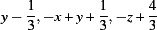.

**Fig. 1.**
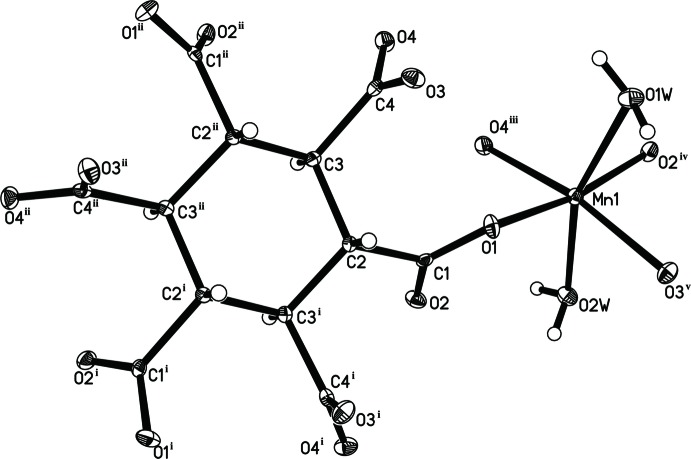
A view of the molecule of (I). Displacement ellipsoids are drawn at the 30% probability level. (i = 1 - x + y,1 - x,z; ii = 1 - x,x-y,z; iii = 1 - x,1 - y,1 - z; iv = y,1 - x + y,1 - z; v = 2/3 + x-y,1/3 + x,4/3 - z)

**Fig. 2.**
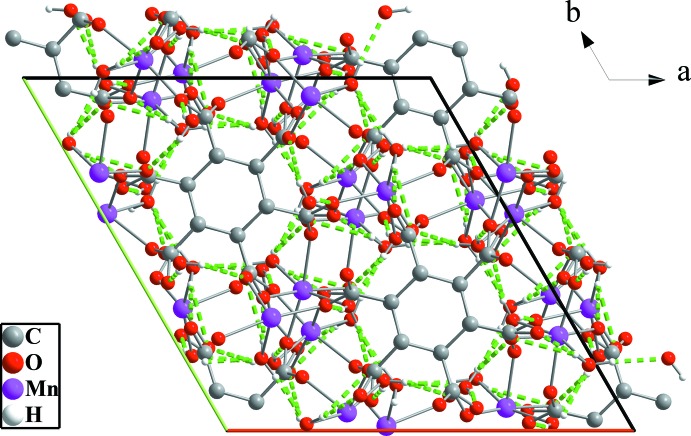
A view along the c axis of the crystal packing of the title compound, hydrogen bonding interactions (dashed lines) in the whole three-dimensional framework.

## supplementary crystallographic information

### Comment

Metal-organic frameworks (MOFs) are an emerging class of periodic crystalline solid-state materials constructed from metal ions or polynuclear metal-oxygen clusters and multidentate organic ligands (Wang *et al.* 2010; Bourne *et al.* 2001). The potential applications in the realm of catalysis, gas separation, luminescence, as well as their intriguing nature of molecular architectures and topologies make so many chemists devote themselves to this active area (O'Keeffe *et al.* 2000; Song *et al.* 2012). The nature of the organic ligand has thus played an important role in designing special metal-organic frameworks. Herein, bicyclo[2.2.2]oct-7-ene-2,3,5,6-tetracarboxylic dianhydride was oxidized and hydrolyzed to give cyclohexacarboxylate anion *in situ*, which exhibits strong coordination ability to ligate the metal atoms.

In this paper, we describe synthesis and the crystal structure of novel three-dimensional Mn^II^-organic compound bearing the ligand 1,2,3,4,5,6-cyclohexacarboxylic acid. X-ray diffraction analysis reveals that the title compound crystallizes in the space group *R*-3. The asymmetric unit contains one crystallographically unique manganese(II) ion, one third cyclohexacarboxylate anion and two aqua ligands (Fig. 1). The central Mn^II^ ion exhibits the octahedral coordination geometry by six oxygen atoms from aqua ligands and different cyclohexacarboxylate anions. The whole framework composed of Mn ions and cyclohexacarboxylate anions is further stabilized by abundant and strong hydrogen bonding interactions (Fig. 2). The hydrogen bonding parameters are listed in Table 1.

### Experimental

A mixture of bicyclo[2,2,2]oct-7-ene-2,3,5,6-tetracarboxylic dianhydride (0.1 mmol, 0.025 g), manganese chloride tetrahydrate (0.1 mmol, 0.02 g) were mixed with deionized water (6 ml) in a 15 ml Teflon-lined reactor, and heated at constant 433 K for 3 d. Then, the mixture was cooled to room temperature at a rate of 5 K h-1. Finally, needle-like crystals were obtained in 27% yield based on MnCl_2_.

### Refinement

All the hydrogen atoms attached to carbon atoms were placed in calculated positions and refined as the riding model, and the water hydrogen atoms were located from the difference maps.

### Crystal data

**Table:**

[Mn_3_(C_12_H_6_O_12_)(H_2_O)_6_]	*D*_x_ = 2.239 Mg m^−^^3^
*M**_r_* = 615.09	Mo *K*α radiation, λ = 0.71073 Å
Trigonal, *R*3	Cell parameters from 11080 reflections
Hall symbol: -R 3	θ = 1.0–25.0°
*a* = 14.5432 (4) Å	µ = 2.15 mm^−^^1^
*c* = 14.9445 (10) Å	*T* = 185 K
*V* = 2737.4 (2) Å^3^	Needle, colorless
*Z* = 6	0.25 × 0.18 × 0.16 mm
*F*(000) = 1854	

### Data collection

**Table:**

Bruker APEXII CCD diffractometer	1200 independent reflections
Radiation source: fine-focus sealed tube	1098 reflections with *I* > 2σ(*I*)
Graphite monochromator	*R*_int_ = 0.025
φ and ω scans	θ_max_ = 26.1°, θ_min_ = 2.1°
Absorption correction: multi-scan (*SADABS*; Bruker, 2001)	*h* = −17→17
*T*_min_ = 0.616, *T*_max_ = 0.725	*k* = −11→17
5063 measured reflections	*l* = −18→18

### Refinement

**Table:**

Refinement on *F*^2^	Primary atom site location: structure-invariant direct methods
Least-squares matrix: full	Secondary atom site location: difference Fourier map
*R*[*F*^2^ > 2σ(*F*^2^)] = 0.025	Hydrogen site location: inferred from neighbouring sites
*wR*(*F*^2^) = 0.064	H atoms treated by a mixture of independent and constrained refinement
*S* = 1.08	*w* = 1/[σ^2^(*F*_o_^2^) + (0.0259*P*)^2^ + 11.1009*P*] where *P* = (*F*_o_^2^ + 2*F*_c_^2^)/3
1200 reflections	(Δ/σ)_max_ = 0.001
112 parameters	Δρ_max_ = 0.75 e Å^−^^3^
4 restraints	Δρ_min_ = −0.28 e Å^−^^3^

### Special details

**Table:**

Geometry. All e.s.d.'s (except the e.s.d. in the dihedral angle between two l.s. planes) are estimated using the full covariance matrix. The cell e.s.d.'s are taken into account individually in the estimation of e.s.d.'s in distances, angles and torsion angles; correlations between e.s.d.'s in cell parameters are only used when they are defined by crystal symmetry. An approximate (isotropic) treatment of cell e.s.d.'s is used for estimating e.s.d.'s involving l.s. planes.
Refinement. Refinement of *F*^2^ against ALL reflections. The weighted *R*-factor *wR* and goodness of fit *S* are based on *F*^2^, conventional *R*-factors *R* are based on *F*, with *F* set to zero for negative *F*^2^. The threshold expression of *F*^2^ > σ(*F*^2^) is used only for calculating *R*-factors(gt) *etc*. and is not relevant to the choice of reflections for refinement. *R*-factors based on *F*^2^ are statistically about twice as large as those based on *F*, and *R*- factors based on ALL data will be even larger.

### Fractional atomic coordinates and isotropic or equivalent isotropic displacement parameters (Å^2^)

**Table:**

	*x*	*y*	*z*	*U*_iso_*/*U*_eq_	
C1	0.72468 (17)	0.55633 (17)	0.56658 (15)	0.0119 (5)	
C2	0.69763 (17)	0.44523 (17)	0.59739 (16)	0.0124 (5)	
H2	0.6975	0.4445	0.6643	0.015*	
C3	0.58420 (17)	0.36429 (17)	0.56474 (16)	0.0126 (5)	
H3	0.5819	0.3650	0.4979	0.015*	
C4	0.50129 (18)	0.38891 (17)	0.60281 (16)	0.0139 (5)	
O1	0.68842 (14)	0.60269 (13)	0.61426 (11)	0.0181 (4)	
O2	0.77520 (13)	0.59359 (13)	0.49548 (12)	0.0180 (4)	
O1W	0.53786 (15)	0.68488 (15)	0.66743 (13)	0.0228 (4)	
O3	0.50339 (13)	0.40393 (14)	0.68557 (11)	0.0190 (4)	
O2W	0.78828 (15)	0.80851 (14)	0.46574 (12)	0.0211 (4)	
O4	0.43295 (13)	0.38944 (13)	0.55035 (11)	0.0187 (4)	
Mn1	0.65353 (3)	0.71812 (3)	0.55736 (2)	0.01313 (13)	
H2A	0.8391 (18)	0.804 (2)	0.4826 (19)	0.020*	
H1B	0.5862 (19)	0.704 (2)	0.7082 (16)	0.020*	
H2B	0.775 (2)	0.786 (2)	0.4129 (13)	0.020*	
H1A	0.4847 (18)	0.6230 (16)	0.6686 (19)	0.020*	

### Atomic displacement parameters (Å^2^)

**Table:**

	*U*^11^	*U*^22^	*U*^33^	*U*^12^	*U*^13^	*U*^23^
C1	0.0076 (10)	0.0110 (11)	0.0147 (12)	0.0029 (9)	−0.0035 (8)	−0.0014 (9)
C2	0.0108 (11)	0.0094 (11)	0.0169 (12)	0.0050 (9)	0.0007 (9)	−0.0006 (9)
C3	0.0100 (11)	0.0102 (11)	0.0165 (12)	0.0043 (9)	−0.0001 (9)	0.0003 (9)
C4	0.0119 (11)	0.0081 (10)	0.0186 (12)	0.0027 (9)	0.0013 (9)	0.0002 (9)
O1	0.0263 (9)	0.0155 (8)	0.0179 (9)	0.0145 (8)	0.0027 (7)	0.0008 (7)
O2	0.0161 (8)	0.0203 (9)	0.0201 (9)	0.0108 (7)	0.0054 (7)	0.0064 (7)
O1W	0.0189 (9)	0.0241 (10)	0.0229 (10)	0.0089 (8)	0.0011 (8)	0.0057 (8)
O3	0.0166 (8)	0.0255 (9)	0.0154 (9)	0.0109 (8)	0.0008 (7)	−0.0015 (7)
O2W	0.0208 (9)	0.0246 (10)	0.0196 (10)	0.0126 (8)	0.0038 (8)	0.0016 (8)
O4	0.0179 (9)	0.0217 (9)	0.0198 (9)	0.0125 (7)	−0.0066 (7)	−0.0052 (7)
Mn1	0.0142 (2)	0.0124 (2)	0.0134 (2)	0.00709 (15)	−0.00102 (13)	0.00031 (13)

### Geometric parameters (Å, º)

**Table:**

C1—O2	1.251 (3)	O2—Mn1^iii^	2.1866 (17)
C1—O1	1.263 (3)	O1W—Mn1	2.2263 (19)
C1—C2	1.530 (3)	O1W—H1B	0.866 (17)
C2—C3^i^	1.540 (3)	O1W—H1A	0.843 (17)
C2—C3	1.550 (3)	O3—Mn1^iv^	2.1581 (17)
C2—H2	1.0000	O2W—Mn1	2.2062 (18)
C3—C4	1.529 (3)	O2W—H2A	0.811 (17)
C3—C2^ii^	1.540 (3)	O2W—H2B	0.838 (17)
C3—H3	1.0000	O4—Mn1^v^	2.1569 (17)
C4—O3	1.254 (3)	Mn1—O4^v^	2.1569 (17)
C4—O4	1.269 (3)	Mn1—O3^vi^	2.1581 (17)
O1—Mn1	2.1565 (16)	Mn1—O2^vii^	2.1866 (17)
			
O2—C1—O1	124.0 (2)	H1B—O1W—H1A	119 (3)
O2—C1—C2	119.9 (2)	C4—O3—Mn1^iv^	131.39 (15)
O1—C1—C2	116.0 (2)	Mn1—O2W—H2A	110 (2)
C1—C2—C3^i^	111.91 (18)	Mn1—O2W—H2B	113 (2)
C1—C2—C3	108.74 (18)	H2A—O2W—H2B	108 (3)
C3^i^—C2—C3	111.7 (2)	C4—O4—Mn1^v^	129.13 (15)
C1—C2—H2	108.1	O1—Mn1—O4^v^	90.49 (6)
C3^i^—C2—H2	108.1	O1—Mn1—O3^vi^	89.74 (7)
C3—C2—H2	108.1	O4^v^—Mn1—O3^vi^	168.84 (7)
C4—C3—C2^ii^	107.13 (18)	O1—Mn1—O2^vii^	171.49 (7)
C4—C3—C2	111.68 (18)	O4^v^—Mn1—O2^vii^	86.09 (6)
C2^ii^—C3—C2	109.3 (2)	O3^vi^—Mn1—O2^vii^	95.12 (6)
C4—C3—H3	109.6	O1—Mn1—O2W	102.94 (7)
C2^ii^—C3—H3	109.6	O4^v^—Mn1—O2W	89.49 (7)
C2—C3—H3	109.6	O3^vi^—Mn1—O2W	79.59 (7)
O3—C4—O4	124.0 (2)	O2^vii^—Mn1—O2W	84.84 (7)
O3—C4—C3	117.0 (2)	O1—Mn1—O1W	89.19 (7)
O4—C4—C3	118.9 (2)	O4^v^—Mn1—O1W	106.90 (7)
C1—O1—Mn1	121.10 (15)	O3^vi^—Mn1—O1W	84.26 (7)
C1—O2—Mn1^iii^	139.24 (15)	O2^vii^—Mn1—O1W	84.36 (7)
Mn1—O1W—H1B	92.6 (19)	O2W—Mn1—O1W	159.65 (7)
Mn1—O1W—H1A	116 (2)		
			
O2—C1—C2—C3^i^	28.8 (3)	C2—C1—O1—Mn1	−151.73 (15)
O1—C1—C2—C3^i^	−154.7 (2)	O1—C1—O2—Mn1^iii^	113.2 (2)
O2—C1—C2—C3	−95.1 (2)	C2—C1—O2—Mn1^iii^	−70.6 (3)
O1—C1—C2—C3	81.4 (2)	O4—C4—O3—Mn1^iv^	16.8 (3)
C1—C2—C3—C4	−60.2 (2)	C3—C4—O3—Mn1^iv^	−160.75 (15)
C3^i^—C2—C3—C4	175.81 (16)	O3—C4—O4—Mn1^v^	−136.2 (2)
C1—C2—C3—C2^ii^	−178.55 (15)	C3—C4—O4—Mn1^v^	41.3 (3)
C3^i^—C2—C3—C2^ii^	57.4 (3)	C1—O1—Mn1—O4^v^	48.74 (18)
C2^ii^—C3—C4—O3	69.9 (3)	C1—O1—Mn1—O3^vi^	−120.11 (18)
C2—C3—C4—O3	−49.8 (3)	C1—O1—Mn1—O2^vii^	115.0 (4)
C2^ii^—C3—C4—O4	−107.7 (2)	C1—O1—Mn1—O2W	−40.85 (18)
C2—C3—C4—O4	132.6 (2)	C1—O1—Mn1—O1W	155.63 (18)
O2—C1—O1—Mn1	24.6 (3)		

Symmetry codes: (i) −*x*+*y*+1, −*x*+1, *z*; (ii) −*y*+1, *x*−*y*, *z*; (iii) *x*−*y*+1, *x*, −*z*+1; (iv) *y*−1/3, −*x*+*y*+1/3, −*z*+4/3; (v) −*x*+1, −*y*+1, −*z*+1; (vi) *x*−*y*+2/3, *x*+1/3, −*z*+4/3; (vii) *y*, −*x*+*y*+1, −*z*+1.

### Hydrogen-bond geometry (Å, º)

**Table:**

*D*—H···*A*	*D*—H	H···*A*	*D*···*A*	*D*—H···*A*
O2*W*—H2*A*···O2*W*^iii^	0.81 (2)	2.31 (2)	3.116 (2)	178 (3)
O2*W*—H2*A*···O3^viii^	0.81 (2)	2.56 (3)	2.955 (2)	111 (2)
O1*W*—H1*B*···O4^vi^	0.87 (2)	1.92 (2)	2.774 (3)	169 (3)
O1*W*—H1*B*···O3^vi^	0.87 (2)	2.52 (3)	2.942 (3)	111 (2)
O2*W*—H2*B*···O1^viii^	0.84 (2)	2.06 (2)	2.883 (3)	169 (3)
O1*W*—H1*A*···O1*W*^iv^	0.84 (2)	2.01 (2)	2.8513 (18)	175 (3)

Symmetry codes: (iii) *x*−*y*+1, *x*, −*z*+1; (iv) *y*−1/3, −*x*+*y*+1/3, −*z*+4/3; (vi) *x*−*y*+2/3, *x*+1/3, −*z*+4/3; (viii) −*y*+4/3, *x*−*y*+2/3, *z*−1/3.
